# Computed tomography in the evaluation of vascular rings and slings

**DOI:** 10.1007/s13244-014-0343-3

**Published:** 2014-07-10

**Authors:** M. Etesami, R. Ashwath, J. Kanne, R. C. Gilkeson, P. Rajiah

**Affiliations:** 1 Cardiothoracic Imaging Section, Radiology, University Hospital of Cleveland, Case Western Reserve School of Medicine, Cleveland, OH USA; 2 Department of Pediatric Cardiology, Rainbow Babies and Children’s Hospital, Cleveland, OH USA; 3 Department of Radiology, University of Wisconsin School of Medicine and Public Health, Madison, WI USA; 4 Cardiothoracic Imaging Section, Department of Radiology, University Hospitals of Cleveland, 11100 Euclid Avenue, Cleveland, OH 44106 USA

**Keywords:** Computed tomography, Vascular rings, Slings, Congenital

## Abstract

Vascular rings are congenital abnormalities of the aortic arch-derived vascular and ligamentous structures, which encircle the trachea and oesophagus to varying degrees, resulting in respiratory or feeding difficulties in children. A sling is an abnormality of the pulmonary arterial system resulting in airway compression. Although several imaging examinations are available for the evaluation of these anomalies, computed tomography (CT) has become the preferred test because of rapid acquisitions, making it feasible to perform the study without sedation or general anaesthesia. Furthermore, CT provides excellent spatial and temporal resolution, a wide field of view, multiplanar reconstruction capabilities and simultaneous evaluation of the airway. In this review, the current role and technique of CT in the evaluation of vascular rings are discussed. A brief discussion of the embryology of the aorta and branch vessels is followed by discussion and illustration of common and some uncommon vascular rings along with critical information required by surgeons.

*Teaching Points*

• *Computed tomography is valuable in the evaluation of vascular rings*.

• *Due to variable clinical and imaging presentations, diagnosis of vascular rings is often challenging*.

• *Laterality of the arch is critical in surgical management*.

## Introduction

Vascular rings are rare (<1 %) congenital abnormalities of the aortic arch-derived vascular or ligamentous structures that encircle the trachea and oesophagus to varying degrees [1]. A sling is an abnormality of pulmonary arterial system resulting in airway compression. Vascular rings and slings may be challenging to diagnose since the clinical presentation is variable and often nonspecific. They can be asymptomatic or present with respiratory symptoms and signs such as respiratory distress, stridor, seal-bark cough, apnoea, cyanosis or recurrent infections, typically in the first year of life. Feeding difficulties such as dysphagia, slow feeding and hyperextension of head while eating may present later in life since liquid diets are tolerated earlier and symptoms typically manifest when solid foods are initiated. Weight loss and failure to thrive may be seen. Some anomalies present later in life with feeding difficulties, during periods of haemodynamic stresses such as pregnancy or when there is ectasia of the vessels. On examination, cough, wheezing, stridor, tachypnoea, noisy breathing and subcostal retractions may be apparent [1]. Vascular rings may be associated with congenital abnormalities, particularly conotruncal anomalies (tetralogy, transposition, truncus) and syndromes including 22q11 deletion [2]. There are no gender, ethnic or geographic predilections [3]. Symptomatic vascular rings are surgically repaired in the first year of life to avoid complications such as hypoxic spells, sudden death, aneurysm, dissection and erosion of the aorta into the trachea or oesophagus.

Imaging plays an important role in the evaluation and management of vascular rings. In this review, the current role and technique of computed tomography (CT) in the evaluation of vascular rings are discussed. A review of the embryology of the aorta and branch vessels is followed by discussion and illustration of common and some uncommon vascular rings along with critical information required by surgeons.

## Imaging modalities in the evaluation of vascular rings

Vascular rings can be identified by several imaging examinations and occasionally multiple imaging tests may be required to make a diagnosis [4]. A radiograph is often the initial imaging test and some abnormalities are found in almost all patients with vascular rings [5]. Arch laterality may be inferred from the anteroposterior (AP) radiograph by the pattern of indentation of the tracheal air column, which is from the right in a right arch, left in a left arch and bilateral in a double arch. On the lateral view, tracheal narrowing may be apparent. Pulmonary hyperinflation may occur with a pulmonary sling. Presence of a right arch with tracheal compression is highly suggestive of a ring. The location of the descending thoracic aorta can be inferred from the paraspinal line and azygo-oesophageal recess. Barium oesophagography is often performed in children with feeding difficulties. The specific type of vascular ring can often be diagnosed based on the pattern of oesophageal indentation on the oesophagram in combination with the pattern of tracheal indentation on the radiograph [6]. Indentation of the posterior oesophagus occurs with a double arch, right arch with an aberrant left subclavian artery or left arch with an aberrant right subclavian artery. Anterior indentation of the oesophagus and posterior indentation of the trachea are caused by pulmonary slings. Bilateral indentation in the AP view is due to a double arch. Right indentation is caused by a right arch or a double arch with left atresia. Left indentation is caused by a double arch with a right arch atresia or circumflex aortic arch with right ductus. Oesophagography does not provide direct visualisation of the ring and hence has been replaced by cross-sectional imaging methods. However, a negative oesophagram excludes a ring and can also evaluate other causes of feeding difficulties such as tracheoesophageal fistula, oesophageal atresia, reflux and aspiration. Tracheography with radioopaque contrast was used in the past to determine the tracheal anatomy. Anterior tracheal indentation is caused by a double aortic arch or aberrant brachiocephalic artery. Posterior tracheal indentation results from a pulmonary sling [7]. Bronchoscopy is performed in patients without a clear diagnosis and to exclude other causes of respiratory distress in children such as foreign bodies and subglottic stenosis. It is also useful in patients with a pulmonary sling for evaluation of complete tracheal rings and other congenital airway anomalies. Invasive angiography is no longer routinely performed because of the advent of CT and cardiovascular magnetic resonance imaging (MRI) [8]. Echocardiography has a limited role in the evaluation of vascular rings because of the small field of view, which is even more limited in patients with a poor thymic window or hyperinflated lungs and inability to detect rings without colour flow Doppler or associated airway abnormalities. However, it is useful in the detection of associated congenital abnormalities, which occur in up to 12–30 % of cases [9]. MRI is one of the two commonly used imaging modalities diagnosing and characterising vascular rings. Advantages of MRI include a wide field of view, multiplanar imaging capabilities and adequate spatial resolution to detect vascular ring and associated airway anomalies, without the use of ionising radiation or iodinated contrast material. Disadvantages of MRI include limited availability, the long acquisition times and the need for deep sedation or general anaesthesia in young children, which may involve high risks in patients with airway compromise. In addition, intubation limits tracheal evaluation [10]. Other disadvantages include the need for different imaging sequences for analysis of airway anomalies, lower spatial resolution than CT, the need for gadolinium-based contrast agents and higher cost.

CT has emerged as the preferred imaging examination for the diagnosis and characterisation of vascular rings. It is performed in symptomatic patients with a suspected vascular ring in other imaging modalities to delineate the anatomy and help surgical planning. Advantages of CT include the rapid acquisition time without the need for sedation or general anaesthesia; high spatial and temporal resolution; large field of view; isotropic voxels with multiplanar reconstruction capabilities; and simultaneous evaluation of the vasculature, airways and, to a lesser degree, the oesophagus. The 3D volume-rendered and shaded surface display images can be helpful for surgical planning and depicting the anomalous anatomy. Ionising radiation and the use of potentially nephrotoxic iodinated contrast material are the primary disadvantages.

## CT Technique

CT angiography (CTA) for the evaluation of vascular rings can be performed without any sedation and with quiet breathing since the latest CT scanners have fast gantry rotation times and high z axis coverage, as a result of which artefacts are minimal. One example of the CTA protocol for vascular rings, using a 256-slice MDCT scanner (Brilliance ICT, Philips, Cleveland, OH, USA) is listed in Table 1. Prospective ECG-triggering eliminates motion artefacts, making it useful in the evaluation of associated cardiac anomalies, although this technique is associated with a slightly higher radiation dose compared to non-ECG-gated scans. We use 1–2 ml/kg of iodinated contrast agent at 350 or 370 mg/mL concentrations. A power injector is preferred over hand injection to obtain homogeneous vascular opacification, at a rate of 1–3 ml/s.

**Table 1:** Technical parameters of a vascular ring protocol

Parameters	ECG gated	Non-ECG gated
Gating	Prospective ECG triggered (40 % R-R interval)	None
Scan mode	Axial, step and shoot	Helical
Detector collimation	128 × 0.625 mm, adaptive z collimation	128 × 0.625 mm, adaptive z collimation
Gantry rotation time	270 ms	270 ms
Temporal resolution	135 ms	135 ms
Scan length	Thoracic inlet to diaphragm	Thoracic inlet to diaphragm
Pitch	–	1.375
Tube voltage	80 kV	80 kV
Tube current	0–3 kg–60 mAs/slice 3–6 kg–80 mAs/slice 6–10 kg–100 mAs/slice 10–15 kg–120 mAs/slice	0–3 kg–60 mAs/slice 3–6 kg–80 mAs/slice 6–10 kg–100 mAs/slice 10–15 kg–120 mAs/slice
Reconstructed slice thickness	0.9 mm	0.9 mm
Slice interval	0.45 mm	0.4 mm
Reconstruction algorithm 4	Idose- level 3	Idose- level 3
Contrast	1–2 ml/kg	1–2 ml/kg
Injection rate	1–3 ml/s	1–3 ml/s
Contrast timing	Empirical	Empirical

Hand injection is used when venous access is challenging and only smaller catheters can be placed. Bolus tracking can be used to initiate image acquisition when contrast attenuation in the aortic arch reaches 100 Hounsfield units (HU) above the baseline, but this is associated with a higher radiation dose due to multiple tracker scans. An empirical delay of 12–15 s after contrast initiation for children less than 10 kg or 20–25 s for larger children can be used [11]. The radiation dose can be minimised using low tube voltage, automatic tube current modulation, non-ECG gating or prospective ECG triggering, higher pitch and iterative reconstruction algorithms.

## Embryology

Although the development of vascular rings can be explained by Edwards’ double ring hypothetical model [12], the development of the arch and branch vessels is more complex, and a thorough understanding of this process is imperative for recognising and accurately characterising the variations of vascular rings, which is critical for surgical planning [13]. Development of the great vessels begins at 20–22 days by vasculogenesis, in which networks of endothelial channels are formed by aggregation of angioblasts. These networks fuse to form the dorsal aortae and aortic arches. The lumen is established within these vessels when the small endothelial channels merge into larger channels [14]. Smooth muscle cells of the media are formed from neural crest cells in the arch and mesenchymal cells in the dorsal aorta [15–17].

**Fig. 1:**
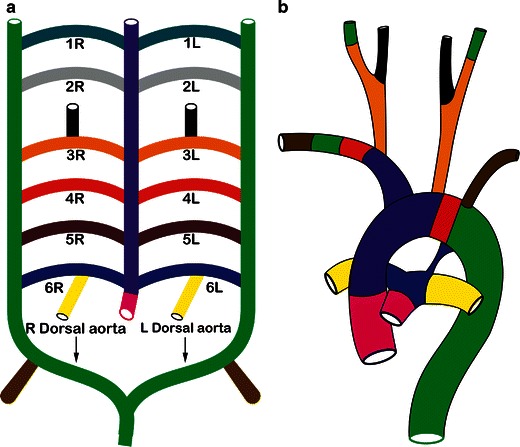
Development of the aorta and branch vessels. **a** Illustration showing the schematic development of the aorta and arch branch vessels from six pairs of aortic arches. Please note that the six arches are never seen together in the foetus, since they develop and regress in a craniocaudal direction. Also, the fifth arch is not present in most foetuses. **b** Illustration showing the fully developed normal left aortic arch and branch vessels, with corresponding colour codes. **a** The left arch between the left common carotid and left subclavian (*red*) is derived from the left fourth arch (*red*). The ventral aorta (*purple*) forms the ascending aorta and the right brachiocephalic trunk while the dorsal aorta (*green*) forms the descending thoracic aorta. The left subclavian artery is derived from the left seventh intersegmental artery (*brown*), while the right subclavian artery is derived proximally from the right fourth arch (*red*), in the mid portion from the right dorsal aorta (*green*) and the distal portion from the right seventh intersegmental artery (*brown*). The aortic root and main pulmonary artery are derived from division of the truncus arteriosus (*pink*). The ductus arteriosus (*blue*) is formed from the dorsal portion of the left sixth arch, while the right and left pulmonary arteries are derived from ventral portions of the right and left sixth arches (*yellow*) respectively. Common carotid arteries are derived from the third arches (*orange*), while internal carotid arteries are formed from the third arches (*orange*) and the dorsal aorta (*green*). External carotid arteries are derived from the third arch branches (*black*)

Six aortic arches are formed in the fourth and fifth weeks of development and run in the centre of the pharyngeal arches connecting the paired ventral and dorsal aortae (Fig. 1a) [18]. The paired ventral aortae fuse to form a single ventral aorta, the aortic sac. Fusion of the dorsal aortae into a single dorsal aorta begins distally and progresses retrograde to the seventh somite. Proximally the aortic sac connects to the heart through the truncus arteriosus. Eventually, the truncus is divided into the ventral aorta and pulmonary trunk by the spiral septum.

The six arches develop in a craniocaudal fashion and also regress in the same fashion; hence all six arches are not seen at the same time (Fig. 1a, b). Also the fifth arch is rarely seen in humans, due to either nonexistence or very early regression [13]. The normal pattern of modelling and regression depends on neural crest cells, although the exact mechanism remains unknown [3, 10, 13, 19].

- The first and second arch vessels form initially and then regress completely by 29 days. The first arch contributes to formation of the maxillary and external carotid arteries, while the second arch contributes to the formation of the hyoid and stapedial branches.
- The third arches persist and form the common carotid, proximal internal carotid and external carotid arteries. The distal internal carotid artery is formed by the dorsal aorta at this level.
- The left fourth arch persists and forms the portion of the left aortic arch between the left common carotid and left subclavian branches. Most of the right fourth arch involutes except for a short proximal portion, which forms the proximal right subclavian artery.
- The fifth arch is either not present in humans or involutes soon after incomplete formation.
- The ventral portion of the right and left sixth arches develops into the proximal right and left pulmonary arteries respectively. The dorsal part of the right sixth arch involutes, but the dorsal part of the left sixth arch persists to form the ductus arteriosus, connecting the distal main pulmonary artery or the left pulmonary artery to the junction of the left fourth arch and the dorsal aorta. The main pulmonary artery develops from the truncus after separation of the conotruncal septum as described above.
- The seventh intersegmental branches of the dorsal aorta contribute to formation of the subclavian arteries. On the left, the dorsal aorta persists for its entire length, but it remodels itself such that the left seventh intersegmental branch forms the left subclavian artery. On the right, the dorsal aorta involutes between its junction with the left dorsal aorta and the origin of the right seventh intersegmental artery. The remaining dorsal aorta between the right fourth arch and seventh intersegmental artery remodels such that the right seventh intersegmental artery forms the distal segment of right subclavian artery. The right dorsal aorta contributes to the mid segment of the right subclavian artery and as described above the right fourth arch forms the proximal segment of the right subclavian artery.
- The ventral aorta forms right and left horns, with the right horn forming the right brachiocephalic artery and the left horn forming the proximal ascending portion of the arch. The brachiocephalic and common carotid arteries elongate because of folding of the embryo.
- Vertebral arteries are formed by anastomosis between seven cervical intersegmental arteries and these lose connection with the dorsal aorta, except at the seventh level, where they form the subclavian arteries.

Abnormalities in this sequential pattern of development and regression result in vascular rings mostly caused by incomplete regression of the distal left fourth arch or an aberrant retroesophageal subclavian artery or a ductus arteriosus originating from the descending thoracic aorta contralateral to the aortic arch.

## Approach to arch anomalies

The normal pattern is a left aortic arch, with a left descending thoracic aorta and a left ductus or ligamentum extending from the proximal descending thoracic aorta to the left pulmonary artery. The arch branches are the right brachiocephalic artery (dividing into the right subclavian and right common carotid), left common carotid artery and left subclavian artery. The different arch types are listed in Table 2, based on Edwards’ hypothetical model [20]. The several subtypes of vascular rings/slings are listed in Table 3. Double aortic arch and right arch with aberrant left subclavian artery account for almost 85–95 % of all symptomatic vascular rings [3].

**Table 2:** Classification of arch anomalies [20]

Group I (Double arch)	Patent bilateral arches	Left ductus Right ductus Bilateral ducti
	Atresia of one arch	Left ductus Right ductus Bilateral ducti
Group 2 (Left aortic arch)	Normal branching	Left ductus Right ductus Bilateral ducti
	Aberrant right subclavian	Left ductus Right ductus Bilateral ducti
Group 3 (Right aortic arch)	Aberrant left subclavian	Left ductus Right ductus Bilateral ducti
	Mirror image branching	Left ductus Right ductus Bilateral ducti

**Table 3:** Various types of vascular rings and slings

Double aortic arch	Right arch dominant Left arch dominant Codominant Atretic smaller arches
Right arch	Aberrant left subclavian artery with Kommerell’s diverticulum and left ligamentum arteriosum/patent ductus arteriosus Mirror image branching with intact retroesophageal ligamentum arteriosum Retroesophageal aortic arch with left descending aorta Right cervical aortic arch with left descending thoracic aorta and aberrant left subclavian artery with patent left ductus arteriosus or intact left ligamentum arteriosum
Left arch	Aberrant right subclavian artery and right ligamentum arteriosum Retroesophageal aortic arch with right descending aorta Left aortic arch, aberrant right subclavian artery, common origin of carotid arteries anterior to the trachea Left cervical aortic arch with right descending aorta, aberrant right subclavian artery and right patent ductus/ligamentum arteriosum
Anomalous innominate artery	
Pulmonary artery sling	
Rare cases	Ductus arteriosus sling Large cervical arch Sagittal compression by ascending and descending aorta Post-arterial switch Situs inversus with left arch and aberrant right subclavian artery and right ligamentum

Evaluation of vascular rings begins with a clear understanding of the definitions of the structures. The aortic arch is the vessel that connects the ascending and descending thoracic aorta and gives rise to arteries supplying the upper extremities and head and neck. Laterality of the arch is defined as the side of the trachea on which the arch crosses either of the main bronchi. Thus a right arch crosses the right main bronchus, a left arch crosses the left main bronchus and a double aortic arch crosses both main bronchi. Sometimes this relationship is not clear, and other clues can be used to establish arch laterality. One rule is that the first arch branch vessel that contains a common carotid artery is contralateral to the aortic arch. For example, if the first branch is the right brachiocephalic artery, which gives rise to the right common carotid artery, the arch is then on the left. However, sometimes the carotid arteries arise close to each other and determining which vessel is the first is a challenge. An exception to the rule is a retroesophageal brachiocephalic artery, which may be the last vessel from the arch, as a result of which the first arch vessel, the right carotid artery, is ipsilateral to the arch. Another rule is that the retroesophageal or aberrant subclavian artery is always contralateral to the arch. However, caution should be exercised when the entire aorta courses posterior to the oesophagus [21].

A vascular ring is defined by encirclement of the trachea and oesophagus by the aorta, arch branch vessels, pulmonary artery, ductus arteriosus or ligamentum arteriosum. A ring is complete or true when there is encirclement on all sides, while it is incomplete or partial when at least one side is not involved. The ring may be caused by patent vascular structures, in which case the diagnosis is usually straightforward. However, identifying the ring can be challenging when atretic vessels or the ligamentum arteriosum are involved. In such cases, clues suggesting the presence of a vascular ring are the “three Ds”: (1) a dimple opposite the arch (ductus); (2) a diverticulum opposite to the arch, which is a fusiform dilation of the ventromedial portion of proximal descending aorta resulting from a remnant to distal segment of embryonic arch [21]; (3) a descending (proximal) aorta contralateral to the arch. Focal narrowing, asymmetry and distortion of the trachea are also indicators. Tracheal compression may occasionally occur without complete encirclement, such as with brachiocephalic artery compression, pulmonary sling, the aorta wrapping around the trachea or orientation of the arch with compression of the main bronchus and right pulmonary artery.

## Double aortic arch

Double aortic arch is the most common symptomatic vascular ring, accounting for 50–60 % of vascular rings [22]. Developmentally it is caused by persistence of both the right and left fourth aortic arches and the right and left dorsal aortae [23]. Double aortic arch usually presents between birth and 3 months, earlier than other symptomatic rings and with more severe symptoms. Occasionally, it can be an incidental finding in older children and adults. There is no association with any major cardiac anomaly. Very rarely it may be associated with tetralogy of Fallot and transposition, but the incidence is not higher than in the general population [23].

**Fig. 2:**
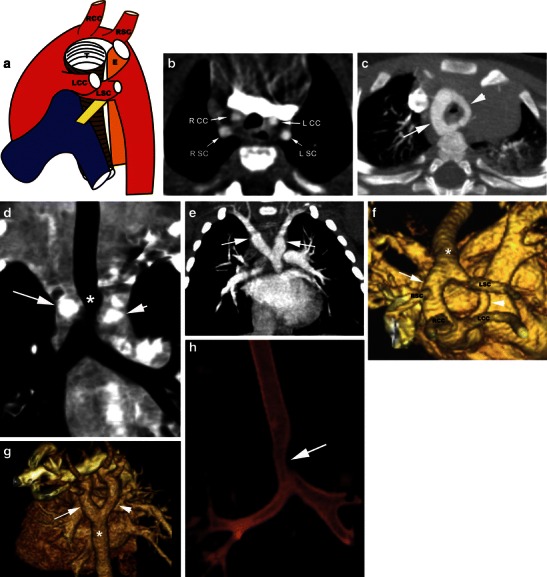
Double aortic arch. **a** Illustration showing the appearances of a double aortic arch, with a dominant right arch. The right arch gives off the right subclavian (*RSC*) and right common carotid (*RCC*) branches, while the left arch gives off the left subclavian (*LSC*) and left common carotid (*LCC*) arteries. Both the trachea and oesophagus are completely encircled and compressed by the vascular ring. **b** Axial CT scan shows the four-vessel sign. The first branch is the right subclavian (*RSC*) artery, the second is the right common carotid artery (*RCC*), the third is the left common carotid (*LCC*) artery and the fourth is the left subclavian (*LSC*) artery. **c** Axial MIP image shows bilateral aortic arches encircling the trachea and oesophagus. The right aortic arch (*arrow*) is larger than the left aortic arch (*arrowhead*). **d** Coronal reconstructed CT image at the level of the trachea shows a larger right arch (*long arrow*) and a smaller left arch (*smaller arrow*) encircling the trachea (*asterisk*). **e** Coronal MIP CT image at the level of the trachea shows similar-sized double arches in another patient (*arrows*). **f** 3D volume-rendered image exquisitely demonstrates the double aortic arch, with a dominant right arch (*arrow*) and a smaller left arch (*arrowhead*), both of which join the descending thoracic aorta (*), which is located on the left. **g** 3D volume-rendered image in another patient (same patient as **e**) demonstrates the double aortic arch, with similar sizes of the right arch (*arrow*) and the left arch (*arrowhead*), both of which join the descending thoracic aorta (*), which is located on the left. **h** Coronal volume-rendered image of the airway shows severe narrowing of the airway (*arrow*) by the double aortic arch

Two arches originate from the ascending aorta, cross on either side of the trachea and oesophagus and join the descending thoracic aorta. The right arch gives rise to the right subclavian and right common carotid arteries, while the left arch gives rise to the left subclavian and left common carotid arteries. The proximal bifurcation of the arches is superior to the level of the distal confluence of arches (Fig. 2a). Usually one arch is dominant and the other arch is smaller or may be atretic (in up to 25–34 % of cases) [3]. The atretic segment is more common in the posterior and distal end of the non-dominant arch. The right arch is dominant in 55–70 % of double arches [22, 24], located more posterior and cephalad than the left arch [25]. Less commonly, the left arch is dominant (20–35 %). In 5–10 % of patients, the arches are equal in size. The descending thoracic aorta is located on the left, but may occasionally be seen on the right or midline. The location of the descending aorta determines the anteroposterior relationship of the arches. If the descending aorta is on the left, as seen in 80 % of patients, the right arch is more posterior than the left arch and crosses to the left to reach descending aorta, but if the descending aorta is on the right, the right arch is located more anterior than the left and the left arch crosses posteriorly to reach the right descending aorta [26]. A ductus arteriosus is usually present when there is no associated intracardiac abnormality. The ductus is usually located on the left, but may be located on the right or very rarely be bilateral [21].

On radiographs, double arches appear as bilateral aortic knobs with midline compression of the trachea. Pulmonary overinflation can result from air trapping. Lateral radiographs show focal tracheal narrowing and anterior bowing. Oesophagography shows bilateral oesophageal indentation on the AP projection and posterior indentation on the lateral projection. On axial CT images, the characteristic appearance of the double arch is the “four-artery sign” (Fig. 2b), referring to a symmetrical trapezoidal or square appearance of the four arch branch vessels at the level of the thoracic inlet. Two arches are seen originating from the ascending aorta and extending on either side of the trachea and oesophagus to form a complete ring. The descending thoracic aorta is located on the left.

Determination of the arch dominance has surgical implications since thoracotomy is performed on the non-dominant side [7]. A single axial image is not always useful in determining which arch is dominant since the ring may not be apparent within a single transverse plane (Fig. 2c). Coronal reformations at the level of the trachea are more useful as they simultaneously depict the arches (Fig. 2d, e). However, the narrowest portion of the arch may not be located at this level. The 3D volume-rendered or surface shaded displays, especially with a left or right posterior oblique view with cranial angulation, are valuable in depicting the ring and sizes of the arches (Fig. 2f, g). In patients with coarctation or hypoplasia of the arch, curved reconstruction along the long axis of the aorta is a good technique. Even in apparently equal-sized aortic arches, one of the arches gets smaller in the posterior aspect near the connection with the descending aorta. If both of the arches are of similar size, the arch with higher flow is the dominant arch. CT can also show the presence and location of the ductus arteriosus, which has to be ligated and divided along with the nondominant arch. Associated airway and oesophageal compression can also be shown (Fig. 2h).

**Fig. 3:**
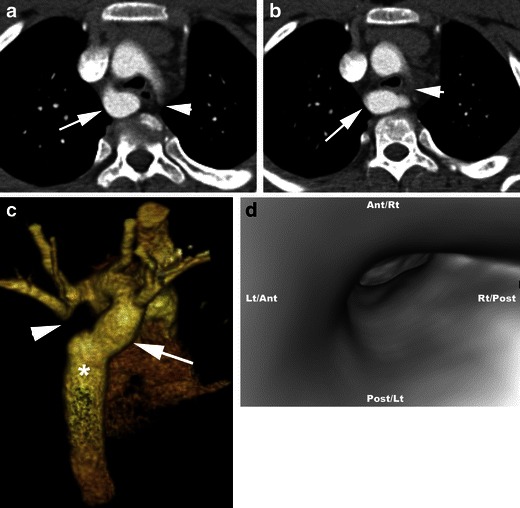
Double aortic arch with an atretic left arch. **a** and **b** Axial CT scan images show bilateral aortic arches. The right aortic arch is dominant (*arrow*). The left aortic arch is atretic (*arrowhead*). **c** 3D volume-rendered image demonstrates the double aortic arch. The dominant right aortic arch (*arrow*) gives off the right subclavian and right common carotid branches, while the left atretic aortic arch gives off the left common carotid and left subclavian arteries proximal to the atretic region (*arrowhead*). Only the right aortic arch joins the descending aorta (*). **d** Virtual bronchoscopy demonstrates severe narrowing of the airway by the double aortic arch with atretic left arch

Double aortic arch with atretic left arch can occur in two settings. First, an atretic segment between the left common carotid and the left subclavian arteries can be confused with a right arch with aberrant left subclavian artery and Kommerell diverticulum. Second, an atretic segment distal to the left subclavian artery can be confused with a right arch with mirror image branching and intact retroesophageal left ligamentum arteriosum (Fig. 3a, b and c). Both forms may be indistinguishable from right aortic arches with a left descending aorta. A clue to the presence of an atretic arch and for distinguishing it from a ligamentum is the tethering and distortion of the left carotid or subclavian artery posteriorly from the aortic arch caused by traction from the atretic arch. Other clues to the presence of an atretic segment include the arterial branching pattern, particularly the posterior course of the proximal head and neck vessels, aortic arch laterality, presence and orientation of a ductal diverticulum and dimple, and focal narrowing of the airway (Fig. 3d) [12, 21]. Even in the absence of these clues, an atretic segment should be suspected with a right aortic arch and retroesophageal diverticulum or right arch with left descending aorta and the surgeon should be alerted. Double aortic arch with an atretic right arch is very rare and has been shown between the right common carotid and right subclavian artery [24].

**Fig. 4:**
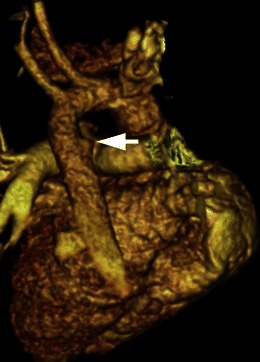
Post-surgical changes of double aortic arch repair. Postoperative sagittal 3D volume-rendered image demonstrates separation of the nondominant left aortic arch (*arrow*) from the descending aorta and surgical interruption of the complete vascular ring

A dominant right aortic arch is repaired using a left thoracotomy, while a dominant left arch is repaired using a right thoracotomy with a muscle sparing approach in the fourth intercostal space. The smaller arch is clamped and then divided near its posterior insertion to the descending thoracic aorta and then the stumps are oversewn. The ligamentum arteriosum is also ligated and divided and any adhesions are released [7] (Fig. 4).

## Right arch-based vascular rings

A right aortic arch occurs in 0.1 % of the population [27]. It is caused by persistence of the right fourth arch and right dorsal aorta and involution of the left fourth arch and dorsal aorta. A right arch begins to the right of the midline and begins descending on the right. At the level of the diaphragm, the descending aorta is on the left, regardless of the laterality of the arch. This transition from right to left is gradual, except for a circumflex aorta (see below). The right main bronchus may be compressed by a sagittally oriented ascending and descending aorta [21]. The typical pattern consists of a right ductus or ligamentum between the proximal descending aorta and the right pulmonary artery, and it is not associated with major intracardiac anomalies. In contrast, the presence of a left ductus or absence of the ductus is associated with major intracardiac anomalies. Tetralogy of Fallot is seen in 30 % of patients [28]. The most common branching patterns of the right arch are the mirror image branching pattern (84 % of cases) and the aberrant left subclavian artery (14 % of cases) [29]. A mirror image branching pattern is associated with cardiac anomalies in 90 % of cases, with tetralogy the commonest abnormality [28].

## Right aortic arch with retroesophageal left subclavian artery, Kommerell’s diverticulum and left ductus arteriosus

**Fig. 5:**
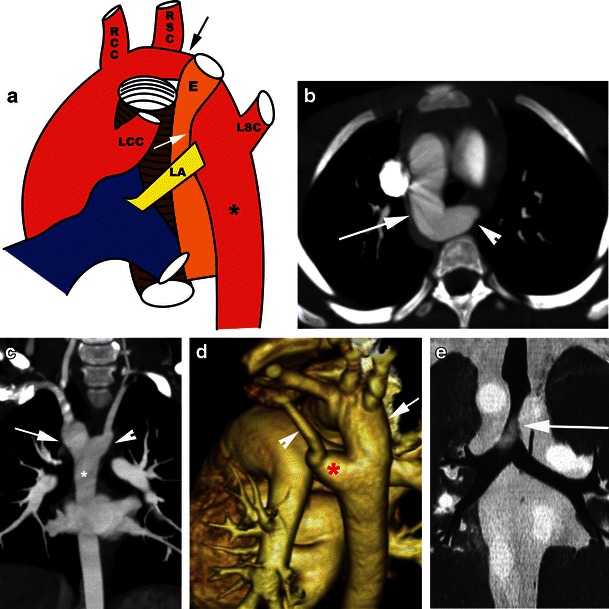
Right aortic arch with Kommerell’s diverticulum. **a** Illustration of a right aortic arch (*black arrow*) with a prominent Kommerell diverticulum (*white arrow*) in the proximal descending thoracic aorta (*), compressing the oesophagus (*E*). **b** Axial *CT* scan shows a right aortic arch (*arrow*) with a large Kommerell diverticulum (*arrowhead*) compressing the posterior oesophagus. **c** Coronal *MIP* image demonstrates a right aortic arch (*arrow*) and prominent Kommerell diverticulum (*arrowhead*) in the proximal descending thoracic aorta (*). **d** Reconstructed 3D volume-rendered image exquisitely demonstrates the right aortic arch (*arrow*) with a large Kommerell diverticulum (*) giving off the retroesophageal aberrant left subclavian artery (*arrowhead*). **e** Coronal CT image shows airway narrowing (*arrow*) caused by the vascular ring

A right arch with an aberrant left subclavian artery is caused by a persistent right fourth arch and regression of the left fourth arch in between the left common carotid and left subclavian arteries. The branching pattern is the left common carotid, right common carotid and right subclavian arteries followed by an aberrant left subclavian artery, which originates as the last branch from the proximal descending aorta and has a retroesophageal course to reach the left subclavian region (Fig. 5a). In 10 % of cases with a right arch and aberrant left subclavian artery, there is a right ductus, in which case, there is no ring or associated intracardiac defect [30]. On CTA, the calibre of the aberrant left subclavian artery is the same from the origin to termination. Sometimes there may be minimal tapering at the base, but it never extends to the level of the trachea. Indentation on the posterior aspect of the oesophagus may be seen.

However, in 90 % of right arches with aberrant left subclavian arteries, there is a left ductus [30]. A right aortic arch with an aberrant left subclavian artery and Kommerell diverticulum is the second most common cause of a symptomatic vascular ring, accounting for 30 % of these cases [31]. The aberrant left subclavian artery originates in the proximal descending aorta from a bulbous diverticulum and courses behind the oesophagus to reach the left. The diverticulum is an embryological remnant of the left fourth arch that persists because of foetal ductal flow to the descending thoracic aorta through the proximal subclavian artery. The ring becomes complete with an intact left ligamentum arteriosum or ductus, which connects the diverticulum to the left pulmonary artery (Fig. 5a). Patients usually present at between 3 and 6 months of age, but most patients are asymptomatic because the ring is relatively loose.

CTA shows the presence of the right aortic arch, the apex of which is located to the right of the trachea. The first branch from the arch is the left common carotid, followed by right common carotid and then right subclavian arteries. The aberrant left subclavian artery originates from the proximal descending thoracic aorta from a Kommerell diverticulum and then has a retroesophageal course to the left (Fig. 5b). There is an abrupt calibre change of the diverticulum, which is always on the side of the aberrant subclavian artery and opposite the side of the arch (i.e. on the left side beyond the level of the trachea), best seen in the coronal plane (Fig. 5c, d). The ductus arteriosus arises from the base of the diverticulum, extending from the descending thoracic aorta to the left pulmonary artery. Although a ligamentum is usually not visualised, the presence of a diverticulum indicates the presence of a vascular ring. An atretic left arch should be raised as a differential diagnosis in this situation. Tracheal compression is caused by the left ductus (Fig. 5e). Occasionally the Kommerell diverticulum enlarges and can independently compress the trachea and oesophagus. This aneurysm may rupture [12].

A right aortic arch with a retroesophageal left subclavian artery, Kommerell diverticulum and left ductus arteriosus is repaired using a muscle-sparing left thoracotomy. The ligamentum arteriosum is ligated or clamped, then divided, and the stumps are oversewn. If the patient also has a large Kommerell diverticulum (1.5 to 2.0 times the normal size), it is resected and the left subclavian artery is then anastomosed to the left common carotid artery so that it does not cause any compression [7].

## Right aortic arch with mirror image branching and an intact retroesophageal left ligamentum arteriosum

**Fig. 6:**
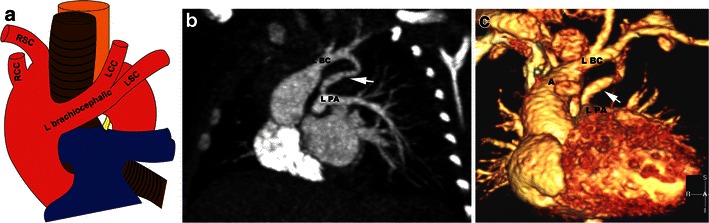
Right arch with mirror image branching and a left ductus arteriosus. **a** Schematic illustration of a right aortic arch with mirror image branching shows a left brachiocephalic artery giving a branch to the left common carotid and left subclavian arteries. **b** Coronal MIP image and **c** Volume-rendered 3D image in a patient with a right aortic arch (*A*) and mirror image branching pattern show a patent ductus arteriosus (*white arrow*) extending from the left brachiocephalic (*L BC*) artery to the left pulmonary artery (*L PA*) forming a ring

A right aortic arch with a mirror image branching pattern is caused by a persistent right fourth arch and partial regression of the fourth left arch between the left subclavian artery and the dorsal aorta. The arch branching pattern is: left brachiocephalic artery (dividing into the left common carotid and left subclavian arteries), right common carotid artery and then right subclavian artery (Fig. 6a). The descending aorta is on the right. The ductus is located on the right in 25 % of these cases, which is not associated with a ring or intracardiac defect [12]. Occasionally the ductus is bilateral, which may or may not be associated with a cardiac defect. An absent ductus is associated with a major intracardiac anomaly. The ductus is located on the left in 75 % of cases, which is almost always associated with a major intracardiac anomaly. The left ductus more commonly extends from the left brachiocephalic artery to the left pulmonary artery, and in such a situation, a ring is not formed [12, 28]. However, a complete ring is formed in a right arch with a mirror imaging pattern when there is a left ductus extending from the proximal descending thoracic aorta and it has a retroesophageal course to the left and then inferiorly to connect to the left pulmonary artery. There is a 90 % association with intracardiac defects, of which tetralogy is the most common [28].

On CTA, there is a right arch with mirror image branching pattern. If there is a patent ductus arteriosus, it can be seen originating from the proximal right descending thoracic aorta or left brachiocephalic artery, crossing behind the oesophagus to connect to the left pulmonary artery (Fig. 6b, c). If there is only a ligamentum arteriosum, the only clue to the presence of this anomaly is a small ductus dimple in the right descending aorta that points to the left, reflecting the residual aortic ductal ampulla. The differential diagnosis for this abnormality is a double aortic arch with an atretic left arch, although in such scenario it is more common to see a left descending aorta in a double aortic arch.

A right arch with retroesophageal left brachiocephalic artery is a rare anomaly, which is not a ring. In this, the branching pattern is a right carotid artery, followed by a right subclavian artery and then an anomalous left brachiocephalic artery arising from an aortic diverticulum, which gives rise to the left common carotid and left subclavian arteries [32]. This is an exception to the rule that the first branch vessel containing the carotid artery is opposite to the side of the arch. This may create an indentation on the posterior aspect of the oesophagus, but is not a complete ring, since there is no structure on the left to complete the ring [21].

## Circumflex retroesophageal right aortic arch

**Fig. 7:**
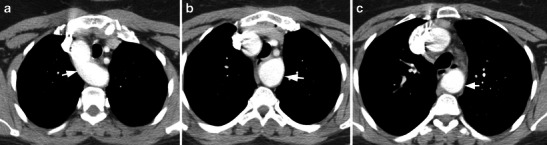
Circumflex right aortic arch. **a** Axial CT image shows a right aortic arch (*arrow*), which extends behind the oesophagus to reach the side. **b** Axial CT image at a lower level shows the arch has now reached the right (*arrow*) and continues as the descending aorta on the left. **c** Axial CT image at a lower level shows the left descending thoracic aorta (*arrow*)

A circumflex retroesophageal aortic arch is the third most common type of vascular ring and occurs when a portion of the aortic arch (either right or left) extends behind the oesophagus while the ascending and descending thoracic aortic segments are located on either side of the spine [33]. In patients with a right arch, the arch runs to the right of the trachea, after which it abruptly courses behind the oesophagus (above the level of the carina) to reach the left where it continues as the descending thoracic aorta (Fig. 7). The branching pattern can be that of an anomalous left subclavian artery or a mirror image branching pattern. The ductus or ligamentum extends from the left descending aorta to the left pulmonary artery, completing the ring. The differential diagnosis is a double aortic arch with an atretic left arch. A common error is to confuse this with a right aortic arch with a left descending thoracic aorta. However, in this variant the crossing is very gradual and happens close to the level of the diaphragm, unlike the abrupt, supracarinal crossing of the circumflex arch.

A circumflex aorta is treated with a median sternotomy and cardiopulmonary bypass. The retroesophageal arch is mobilised, divided and brought anterior to the airway and anastomosed end to side with the lateral portion of the ascending aorta. Most of these patients have division of the left ligamentum through a left thoracotomy [7].

## Left arch variants

### Left aortic arch with aberrant right subclavian artery and right ligamentum arteriosum and right descending aorta

**Fig. 8:**
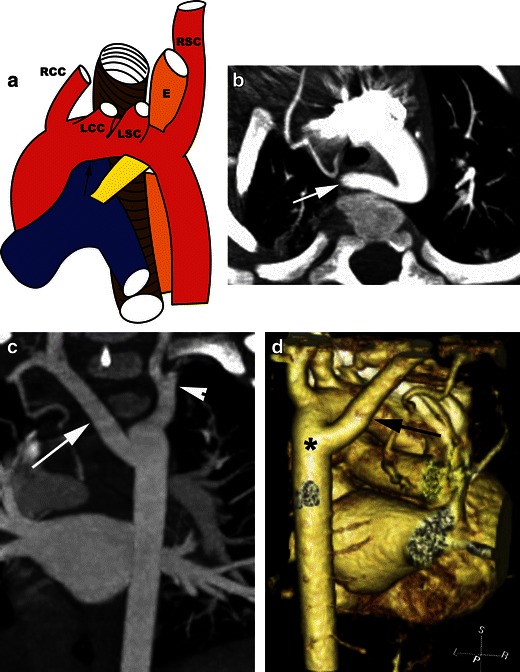
Left aortic arch with an aberrant right subclavian artery. **a** Illustration showing the appearances of a left aortic arch (*arrow*) with an aberrant right subclavian (*RSC*) artery. The first branch from the arch is the right common carotid (*RCC*) artery, followed by the left common carotid (*LCC*) artery and then the left subclavian (*LSC*) artery. The right subclavian (*RSC*) artery has an aberrant origin as the last branch originating from the proximal descending thoracic aorta and has a retroesophageal (*E*) course to reach the right side. **b** Axial CT scan shows an aberrant right subclavian artery (*arrow*) originating from the proximal descending thoracic aorta and has a retroesophageal course to reach the right. Posterior indentation and compression of oesophagus are seen. **c** Coronal MIP image demonstrates the aberrant right subclavian artery (*arrow*) originating from the proximal descending aorta and reaching the right. Normal left subclavian artery (*arrowhead*) is also seen. **d** Coronal reconstructed 3D volume-rendered image shows the aberrant right subclavian artery (*arrow*) originating from the proximal descending thoracic aorta (*) and coursing to the right behind the oesophagus, which is compressed

A left aortic arch with an aberrant right subclavian artery is the most common vascular abnormality of the aortic arch, occurring in 0.5 % of the population [20]. This anomaly is caused by regression of the right arch between the right subclavian and right common carotid arteries. The right subclavian artery then inserts into the proximal descending thoracic aorta. The arch vessel branching pattern is the right common carotid, left common carotid, left subclavian and right subclavian arteries (Fig. 8a). This is usually associated with a left ductus and hence a ring is not produced since there is vasculature on only three sides of the trachea and oesophagus. Although this does not produce a vascular ring, sometimes there may be minimal posterior compression of the oesophagus, causing dysphagia lusoria, which is more common in adults than children, presenting in the 4th or 5th decade. Symptoms may however result from dilation, calcification and hardening of the aberrant subclavian artery. Oesophageal manometry may be required to decide whether compression is the cause of symptoms in these patients.

**Fig. 9:**
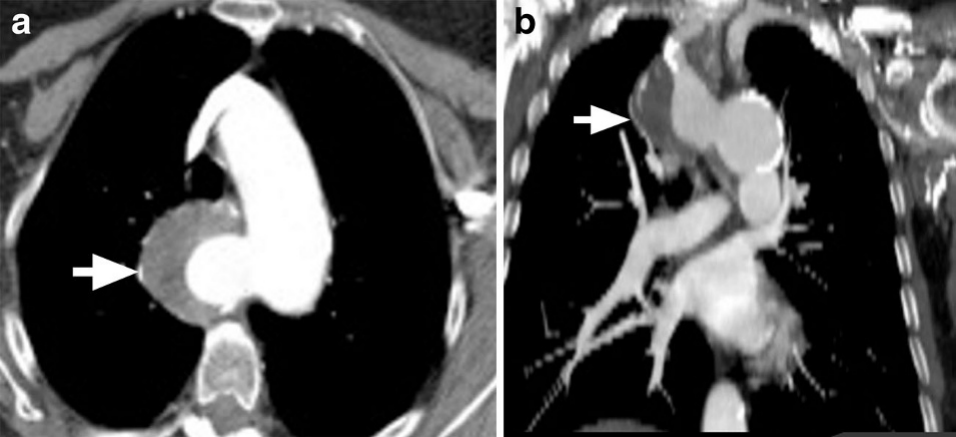
Aneurysm of an aberrant right subclavian artery. **a** Axial CT scan shows a large aneurysm with partial thrombosis (*arrow*) at the origin of an aberrant right subclavian artery in a patient with a left aortic arch. **b** Coronal CT reformatted image in the same patient shows the partially thrombosed aneurysm (*arrow*) at the origin of an aberrant right subclavian artery

However, very rarely a ring may be produced in the left arch with an aberrant right subclavian artery in the presence of a right ductus and a circumflex right descending thoracic aorta [12, 24]. The right ligamentum arteriosum or ductus extends from the aberrant right subclavian artery to the right pulmonary artery, forming a vascular ring [12]. On CTA there is a left aortic arch. The first branch is the right common carotid artery, followed by the left common carotid and left subclavian arteries. The right subclavian artery originates as the last branch from the proximal descending thoracic aorta and then courses behind the oesophagus to reach the right (Fig. 8b, c and d). The origin of the aberrant vessel may be dilated, the Kommerell diverticulum. A ring is present if there is an associated right ductus/ligamentum. Aneurysms may be seen (Fig. 9a, b).

### Circumflex left aortic arch

In this anomaly, the arch courses to the left of the trachea, then extends behind the oesophagus to reach the right and continue as the right descending aorta. A ring is formed when a right ductus or ligamentum connects the descending aorta to the right pulmonary artery. The branching pattern is that of a left arch with an aberrant right subclavian artery. Occasionally a three-vessel branching pattern is seen in which there is no ring.

### Brachiocephalic artery compression

**Fig. 10:**
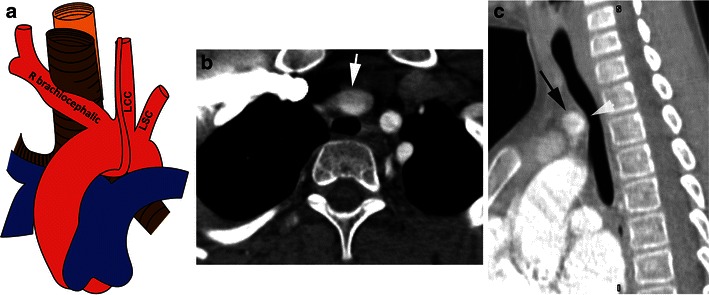
Anomalous innominate artery. **a** Schematic illustration shows an anomalous course of an innominate artery originating more posterior and to the left than normal, coursing to the right anterior to the trachea. **b** Axial CT image shows a dilated innominate artery (arrow) compressing the anterior trachea. **c** Sagittal reformatted MinIP (minimal intensity projection) image shows indentation of the anterior aspect of the trachea (*arrowhead*) by a dilated anomalous innominate artery (*black arrow*)

In this anomaly, the brachiocephalic artery has an anomalous course, originating more posterior and to the left, from the aortic arch, resulting in anterior tracheal compression as the artery extends to the right superiorly and posteriorly to reach the right subclavian region (Fig. 10a) [34]. On CTA, the origin of the anomalous brachiocephalic artery (Fig. 10b) as well as anterior indentation of trachea (Fig. 10c) is demonstrated. Bronchoscopy shows a pulsatile mass compressing the anterior trachea from the left to right at the level of the vocal cords, which is much higher than the other vascular rings. Compression becomes significant when 70–80 % of the tracheal lumen is compromised [7]. During bronchoscopy, a diminished right pulse can be demonstrated if the anterior trachea is compressed by the scope. This anomaly is surgically treated through a right anterolateral thoracotomy (third interspace), lifting the brachiocephalic artery away from the anterior tracheal wall and suspending it to the posterior aspect of the sternum. An alternative technique is to perform a median sternotomy, divide the brachiocephalic artery and reimplant it at a more anterior and right position in the ascending aorta [7].

### Pulmonary artery sling

**Fig. 11:**
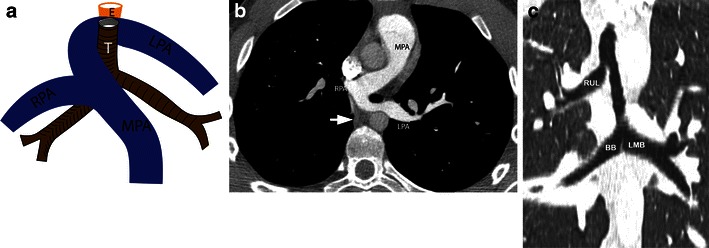
Pulmonary sling. **a** Illustration showing an anomalous left pulmonary artery (*LPA*), which originates from the right pulmonary artery (*R PA*) and then crosses between the trachea (*T*) and oesophagus (*E*) to reach the left. **b** Axial CT scan shows an anomalous left pulmonary artery (*L PA*), which originates from the right pulmonary artery (*R PA*) and then crosses between the trachea and oesophagus (*white arrow*) to reach the left. **c** Coronal MinIP image of the airway shows a bridging bronchus (*BB*) that originates from the left main bronchus (*LMB*) and crosses the mediastinum to reach the side where it supplies the right middle and lower lobe. The right upper lobe (*RUL*) bronchus arises from the trachea

Pulmonary artery sling is characterised by the left pulmonary artery arising from the right pulmonary artery and then passing over the right main bronchus and between the trachea and oesophagus to reach the left hilum (Fig. 11a). In this process, it forms a sling that compresses the trachea and oesophagus. This anomaly is caused by failure of the development or obliteration of the left sixth aortic arch when the developing left lung bud captures its vascular supply from the right sixth arch, caudal to the developing tracheobronchial tree [35]. A pulmonary sling may also be associated with several airway anomalies including compression of the trachea and right main bronchus by the anomalous artery, complete tracheal rings (ring-sling complex) [36] and tracheobronchomalacia. A pulmonary sling is classified according to the associated airway anomalies [37].

Type I demonstrates compression of the trachea and right main bronchus by the anomalous pulmonary artery, but the airway branching is normal. Type I is associated with lower morbidity and mortality [37]. Type IA has no associated airway abnormality. Type IB is associated with the tracheal bronchus, tracheobronchomalacia and unilateral pulmonary hyperinflation.

In type II the anomalous pulmonary artery is more caudal and associated with long segment tracheobronchial stenosis. Associated anomalies include tracheobronchial branching abnormalities, left intermediate and right bridging bronchi, low inverted T-shaped carina, complete tracheal rings and bilateral pulmonary hyperinflation [37].

CTA shows the anomalous origin of the left pulmonary artery from the right pulmonary artery after which it courses between the trachea and oesophagus to reach the left (Fig. 11b). The differential diagnosis for a pulmonary sling is right pulmonary agenesis, where the right pulmonary artery is absent and the left pulmonary artery originates from the main pulmonary artery. Airway anomalies are also demonstrated on CT. With complete tracheal rings, the posterior membrane is absent, and there are circumferential tracheal cartilages. The airway appears round and narrow, as small as 2–3 mm, with a lack of change of caliber between inspiration and expiration. With a bridging bronchus anomaly, the right middle and lower lobes are supplied by a bronchus that originates from the left main bronchus, which bridges the mediastinum to reach the right. The right upper lobe is supplied by right main bronchus originating from the trachea at the level of the carina (Fig. 11c) [38]. Other variants of this branching exist [39]. With tracheobronchomalacia, there is severe (>50 %) narrowing of the airway in expiration.

A pulmonary sling is managed by a median sternotomy and cardiopulmonary bypass. The anomalous left pulmonary artery is ligated, divided and then reimplanted into the main pulmonary artery anterior to the trachea. If there are associated complete tracheal rings, these can be repaired using tracheal resection or end-to-end anastomosis, tracheal autograft, pericardial patch tracheoplasty or slide tracheoplasty [7].

## Uncommon rings

There are several uncommon types of vascular rings. A right cervical aortic arch with an aberrant left subclavian artery, left descending aorta and left ductus/ligamentum is a rare anomaly with the aortic arch developing from the third instead of the fourth arch [40]. Similarly, the left cervical aortic arch with an aberrant right subclavian artery, right descending thoracic aorta and right ductus or ligamentum can also produce a ring [12, 40]. A left aortic arch, aberrant right subclavian artery and common origin of the carotid arteries anterior to the trachea can cause tracheal and oesophageal compression between the two large vessels [12]. The presence of both the ascending and descending thoracic aorta in the same anteroposterior plane is an unusual cause of tracheal compression [21]. A large left cervical arch may cause anterior tracheal compression, even without a right ductus, which is treated with a right thoracotomy [21, 41]. A ductus arteriosus sling is a rare anomaly in which the ductus arteriosus extends from the right pulmonary artery to the proximal descending thoracic aorta between the trachea and oesophagus, with an associated aberrant right subclavian artery that compresses the trachea and right bronchus (similar to a pulmonary sling) [42]. Other rare vascular rings include a right aortic arch with a right ligamentum and absent left pulmonary artery [43], situs inversus with a left arch, aberrant right subclavian artery and right ligamentum [7] and compression by the pulmonary artery following arterial switch repair [41]. A persistent fifth aortic arch is an extremely rare anomaly, which however does not cause a vascular ring. Arches derived from the fourth and fifth foetal arches are present to varying degrees [44].

## Conclusion

Vascular rings are complex and diagnosis is often challenging because of variable and non-specific clinical presentations. CT angiography plays an important role in the identification and definition of the anatomy of these complex anomalies, thus providing a roadmap to surgeons. Careful analysis of the arch laterality, branching pattern and position of the ductus or ligamentum is essential for accurate characterisation. Associated airway anomalies are also assessed using CT.

## Abbreviations

MRI: Magnetic resonance imaging
CT: Computed tomography
CTA: CT angiography
